# Relationship between the Polymeric Ionization Degree and Powder and Surface Properties in Materials Derived from Poly(maleic anhydride-*alt*-octadecene)

**DOI:** 10.3390/molecules23020320

**Published:** 2018-02-02

**Authors:** Constain H. Salamanca, Cristhian J. Yarce, Camilo A. Zapata, Jonnathan A. Giraldo

**Affiliations:** 1 Programa de Maestría en Formulación de Productos Químicosy Derivados, Facultad de Ciencias Naturales, Universidad Icesi, Calle 18 No. 122–135, Cali 760031, Colombia; cjyarce@icesi.edu.co; 2 Departamento de Ciencias Farmacéuticas, Facultad de Ciencias Naturales, Universidad Icesi, Calle 18 No. 122–135, Cali 760031, Colombia; cazg15@hotmail.com (C.A.Z); jhonatan.a24@hotmail.com (J.A.G.)

**Keywords:** poly(maleic anhydride-*alt*-octadecene), anionic polyelectrolyte, ionization degree, humidity loss and gain, powder flowability

## Abstract

Polymeric materials derived from poly(maleic anhydride-*alt*-octadecene)—here referred as PAM-18—have shown interesting properties that make them potential pharmaceutical excipients. In this work, eight polymers derived from PAM-18 were obtained using NaOH and KOH at 1:1; 1:0.75, 1:0.5, and 1:0.25 molar ratios. The resulting products were labeled as PAM-18Na and PAM-18K, respectively. Each polymer was purified by ultrafiltration/lyophilization, and the ionization degree was determined by potentiometric studies, which was related to the zeta potential. The structural characterization was performed using the Fourier transform infrared (FT-IR) espectroscopy, differential scanning calorimetry (DSC), thermogravimetric analysis (TGA), and X-ray diffraction (XRD) techniques. The physical characterization was carried out by SEM, particle analysis, and humidity loss and gain studies; the surface studies were performed by the sessile drop method. PAM-18Na had ionization degrees of 95%, 63%, 39% and 22%, whereas those for PAM-18K were 99%, 52%, 35% and 20%, respectively. The results also showed that for higher inorganic base amounts used, the polymeric materials obtained possess high ionization degrees, which could form polymeric solutions or hetero-dispersed systems. Likewise, it was observed that for higher proportions of carboxylate groups in the polymeric structure, the capability to retain water is increased and, only can be eliminated by drying at temperatures greater than 160 °C. On the other hand, the modification of PAM-18 to its ionized forms led to the formation of powder materials with low flowability and surfaces that ranged from very hydrophobic to slightly wettable.

## 1. Introduction

**Figure 1:**
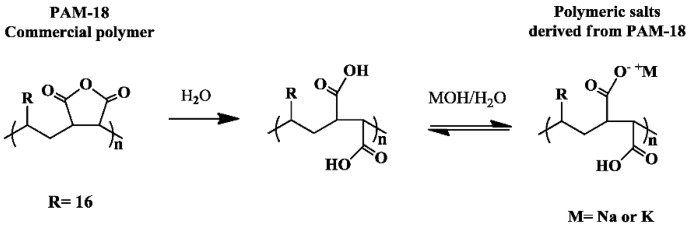
Scheme of the ionization process of polymers derived from poly(maleic anhydride-*alt*-octadecene) (PAM-18).

Poly(maleic anhydride-*alt*-octadecene)—also named as PAM-18—is a copolymeric material usually obtained by free radical polymerization reaction between maleic anhydride and vinyl-octadecene [1,2]. This PAM-18 polymer displays both hydrophobic and hydrophilic behavior due to the alkyl chain and the anhydride group, respectively. One of the most interesting properties of PAN-18 is its capacity to generate anionic polyelectrolytes in aqueous media, when the maleic anhydride group is split in to two carboxylic acids, which can be ionized under basic conditions [3,4,5,6], as shown in Figure 1. 

The monomeric composition of PAM-18 allows this polymer to have an ionization degree that depends on the acidity/basicity of the solution. Consequently, polymers derived from PAM-18 might change their physicochemical properties, becoming soluble in aqueous or organic solvents. These polymers could also generate hydrophobic “pseudo-phases” in aqueous media, where it is possible to aggregate molecules acting like a reservoir system [7,8,9,10]. However, it is worth mentioning that few studies report the relationship between the ionization degree and the physicochemical properties of this class of polymers, in solution and the solid state, and in a minor degree for amphiphilic materials that change the hydrophilic/hydrophobic balance according to the carboxylate/carboxylic acid ratio in the polymer backbone. 

Likewise, these copolymers derived from maleic anhydride have shown great potential as controlled drug release systems [11], and have drawn substantial significance due to their biocompatible characteristics, clearly defined structure, and versatility in combination with other precursors, leading to a wide variety of polymeric materials with multiple properties and applications. The most important claims correspond to their ability to act as (i) a surface coating systems [12,13], (ii) rheological modifiers [14], and (iii) nano-stabilizers in heterodisperse media [15].

Other polymeric materials derived from maleic anhydride correspond to the potassium and sodium salts of poly(maleic acid-*alt*-octadecene) and are known as PAM-18K and PAM-18Na, respectively. These salts are the main focus of this study. These materials have shown interesting surface properties, such as the ability to reduce the surface tension of water [16], forming intra- and inter-molecular hydrophobic pseudo-phases in aqueous media [17,18]. The present work focusses on the physical and structural characterization of some polymers derived from PAM-18 with different ionization degrees. We also discuss how the ionization degree leads to changes in different characteristics of pharmaceutical interest, such as the ability to trap or lose water, the ability to flow as a powder material, or the ability to generate hydrophobic surfaces.

## 2. Results and Discussion

### 2.1. Obtention of Polymeric Materials Derived from PAM-18 

It was found that each polymer derived from PAM-18 presented reaction yields higher than 90%, regardless of the inorganic base used. Regarding the ionization process, the polymers presented different behaviors depending on the molar ratio between the inorganic alkali and the co-monomeric units of PAM-18. For ratios 1 and 0.75 (with respect to the PAM-18 co-monomeric unit), the system shifted from a heterogeneous form to a clear solution with yellowish coloration. However, polymers having the lowest molar ratio of 0.50 and 0.25 formed heterodisperse gel-like structures created by pH-dependent polymers, such as those derived from acrylic acid [19,20]. These results are very interesting, considering: (i) the potential applications shown by the PAM-18 polymer (both in its anhydride form [21] and in its hydrolyzed form [22,23]), (ii) the amphiphilic nature of the polymer, which depends on the balance of hydrophilic and hydrophobic groups in the polymeric backbone. This balance allows us to obtain materials with different degrees of hydrophobicity, either for coating in the solid state or for the formation of different kinds of colloidal systems, both lyophilic (polymer solutions) and lyophobic (heterodisperse suspensions) in aqueous media.

It was observed that the freeze-drying times were longer when the polymers had higher ionization degree. This result suggests that such polymers have different ways of interacting with water. In the case of high ionization degrees, the polymers tend to interact more strongly, while at lower ionization degrees, such interactions are weaker, allowing a faster removal of water in the lyophilization process.

### 2.2. Determination of Ionization Degree and Zeta-Potential

Two points of inflection were observed during the potentiometric titrations study, corresponding to the presence of carboxylate and carboxylic acid groups on the polymeric backbone. The results of the ionization degree for PAM-18Na and PAM-18K polymers are summarized in Table 1. In the case of a 1:1 molar ratio between the PAM-18 polymer and the inorganic base (NaOH or KOH), the ionization degree achieved was close to 100%; when the amount of inorganic base decreased, the ionization degrees reached were smaller than expected. This result could be explained considering several aspects of the reaction process: (i) the temperature and the reaction times were the same in each case; (ii) the lower amount of inorganic base led to a lesser degree of polymeric solvation and thus to a lower availability for the ionization process. 

The results of the media pH and zeta-potential values of the polymeric materials in aqueous media are in agreement with the results obtained from the ionization degree. Therefore, a higher ionization degree leads to basic pH values corresponding to a high fraction of carboxylate groups formed in the polymer backbone. On the other hand, low ionization degrees lead to neutral and slightly acidic pH values, indicating a greater amount of carboxylic acid groups than carboxylates in the polymer. Regarding zeta-potential studies, the polymer surface was always negative, and increased as the ionization degree increased, which agrees with the previous results.

### 2.3. Structural Characterization of Polymer Materials 

#### 2.3.1. Fourier Transform Infrared Spectroscopy (FT-IR) Characterization

**Figure 2:**
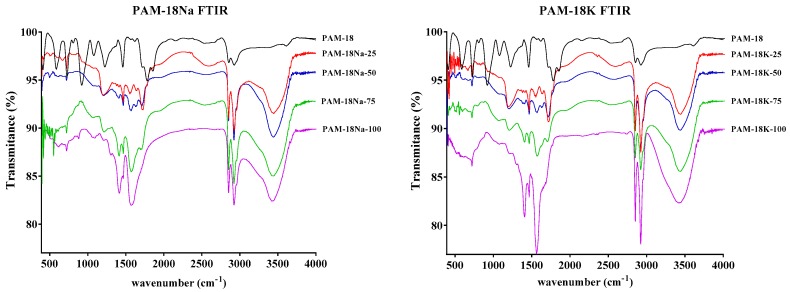
Fourier transform infrared (FT-IR) spectra of polymers derived from PAM-18.

The structural changes of the PAM-18 precursor and PAM-18Na and PAM-18K derivatives were analyzed by comparing the FT-IR spectra (Figure 2). Briefly, the FT-IR spectra of the polymers derived from PAM-18 showed typical signals corresponding to the symmetric and asymmetric stretching values of the alkyl chain at ~2920 and 2848 cm^−1^, respectively. Likewise, the conventional signal of the hydroxyl group from carboxylic acid at ~3110 cm^−1^ was observed, corroborating the presence of carboxylic acid and carboxylate forms in PAM-18Na and PAM-18K. The synthesis of these derivatives is verified by the formation of two signals. Thus, the carbonyl groups in maleic anhydride appeared at ~1773 and 1704 cm^−1^ and shifted to ~1706 and 1556 cm^−1^ upon the formation of the carboxylic acid and carboxylate species [1]. However, polymers having an ionization degree close to 100% showed only two carbonyl group signals between ~1706 and 1556 cm^−1^, ascribed to the carboxylate forms. All other polymers showed four carbonyl group signals (~1750, 1706, 1550, and 1450 cm^−1^) attributed to carboxylic acids and carboxylates.

#### 2.3.2. X-ray Diffraction (XRD) 

**Figure 3:**
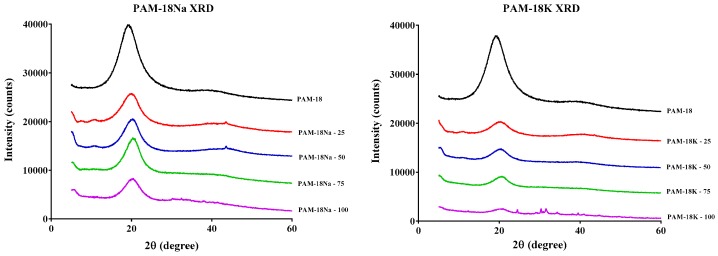
X-ray powder diffractograms of polymers derived from PAM-18.

Figure 3 presents the X-ray powder diffractograms for the polymeric salts derived from PAM-18. Broad dispersion patterns are observed, which is typical of amorphous and atactic polymeric materials [24,25,26]. Additionally, the results show that the hydrolysis/ionization process does not significantly affect the polymeric amorphousness in the materials. Moreover, the X-ray diffraction behavior was very similar in both polymeric families, and the Na^+^ and K^+^ counter ions did not seem to make a significant difference.

#### 2.3.3. Thermogravimetric Analysis (TGA)

**Figure 4:**
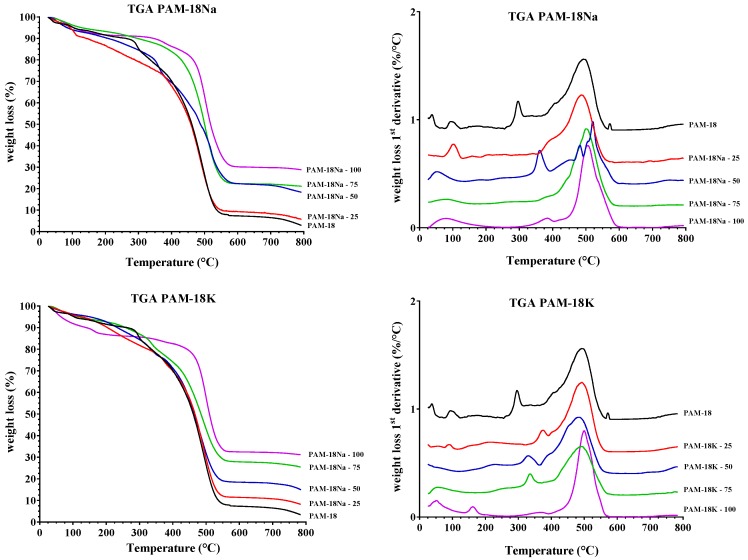
Thermogravimetric analysis (TGA) of polymers derived from PAM-18.

The results of the thermogravimetric analysis are presented in Figure 4. First temperature derivatives of the weight loss are displayed with the aim of highlighting the zones of change in the weight loss with respect to the increase of the temperature. These results showed a loss of weight at around 100 °C, which corresponds to the loss of water associated with the polymeric materials. There was also a considerable change in the weight of the materials between 300 °C and 600 °C, due to the decomposition of the organic material present in the polymers. Inorganic materials began to form above 600 °C, where the quantity of product was more significant as the amount of the Na^+^ and K^+^ counter ions increased in the polymeric materials.

#### 2.3.4. Differential Scanning Calorimetry (DSC) Analysis

**Figure 5:**
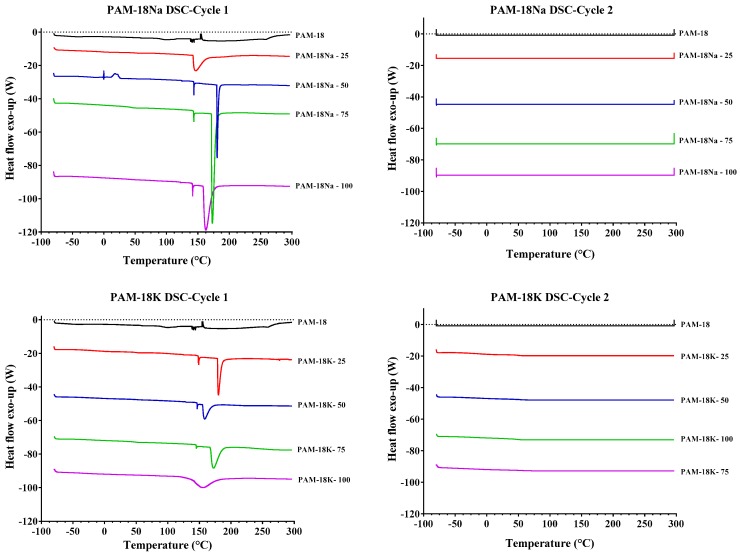
Differential Scanning Calorimetry (DSC) of polymers derived from PAM-18.

The conventional and double heating cycles of DSC assays are presented in Figure 5. The double heating cycle allows confirmation of whether hydrated forms of the polymeric materials are generated in the hydrolysis/ionization process and give further details about those results observed by TGA, related with the weight losses around 80–200 °C. In the case of the PAM-18 polymer (black curves), only one thermal event was observed between −15 °C and 20 °C, which was attributed to a glass transition because this thermal event remained with the second heating cycle. 

In the case of the PAM-18Na-25 and PAM-18K-25 materials (red curves), the same glass transition event was observed along with another endothermic event given between 150 °C and 170 °C (first heating cycle), which disappeared with the second heating cycle. This result indicates that such materials present bound water probably obtained in the hydrolysis/ionization processes. On the other hand, the rest of the polymeric materials showed the same glass transition event given in the precursor material. Likewise, endothermic events related to the loss of bound water in the same temperature range and signal disappearance in the second heating cycle were also observed [27]. Finally, it is worth noting that PAM-18Na materials presented a higher intensity in the endothermic curve than the PAM-18K materials. This result suggests that PAM-18Na materials tend to bind water more strongly than PAM-18K materials.

### 2.4. Characterization of Powder Polymeric Materials 

#### 2.4.1. Powder Morphology and Shape

**Figure 6:**
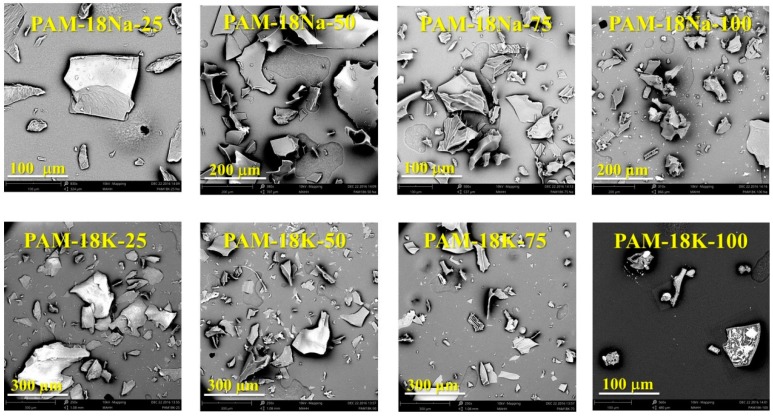
SEM images of polymers derived from PAM-18.

The SEM images of PAM-18 derivatives correspond to amorphous plates which is a typical morphology produced by the lyophilization drying process (Figure 6).

#### 2.4.2. Flowability Assays

The results of the particle study are summarized in Table 2. According to the guidelines established in the USP 40/NF35 [28], the Carr index and Hausner ratio—values between 11 and 23% and 1.2–1.3, respectively—suggest that the flowability of the powder ranged from acceptable to poor, whereas the angle of repose values from 30 to 40 suggest good-to-intermediate flowability properties. Therefore, our results show that these polymers presented intermediate-to-poor flow properties. This result is consistent with the processing method, where polymer plates are collected and subsequently sifted. Moreover, according to the nature of the polymeric salt, these powders tended to absorb water from the environment, leading to changes in their flowability. In order to get an in-depth analysis of such behavior, the humidity loss and gain data are presented and discussed below. 

#### 2.4.3. Humidity Loss and Gain Study

**Figure 7:**
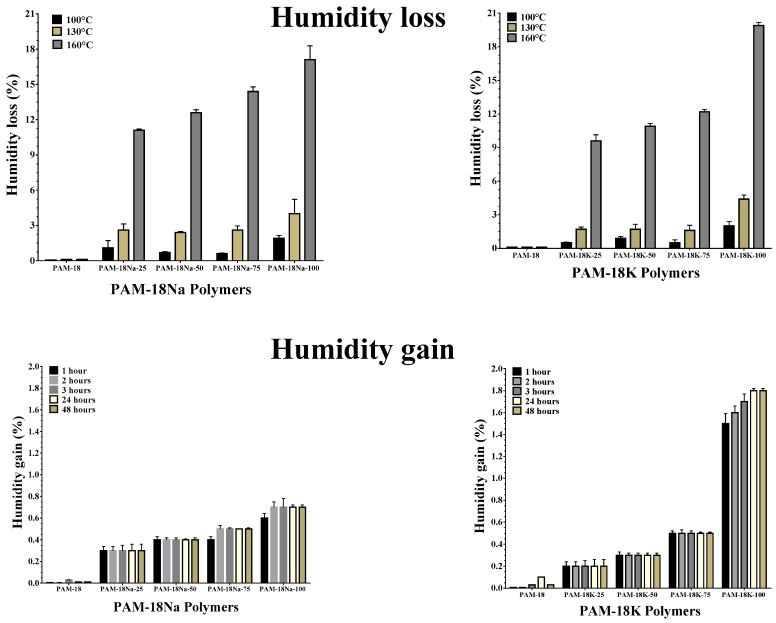
Loss and gain of humidity by the PAM-18Na and PAM-18K polymers.

The capability of the loss and gain of humidity by the PAM-18Na and PAM-18K polymers are shown in Figure 7. The results of the humidity loss show a strong relationship with the results obtained by DSC and TGA, where the increment in the applied temperature led to higher humidity loss in the polymeric materials. This result suggests that different polymeric materials bind water differently. In the case of humidity loss at 100 °C, the results showed that, for the PAM18 precursor polymer, no significant humidity loss was observed, which is consistent due to the hydrophobic nature of the polymer. In the case of the modified materials, a humidity loss lower than 3% was observed for the materials with higher ionization degrees (PAM-18Na-100 and PAM-18Na-100) and less than 1% for the rest of the polymeric materials. These humidity losses could be attributed to the moisture adsorbed on the polymeric powder surface. 

In the case of humidity loss at 130 °C, results like those at T = 100 °C were observed. However, the humidity loss for T = 130 °C was slightly higher than those found at 100 °C. For the PAM-18 polymer, no humidity loss was observed, while for the materials with the highest ionization degree, the humidity loss was around 4% and lower than 3 % for the rest. In the case of moisture loss at 160 ° C, the results are very interesting, and show that an increase in the polymeric ionization degree led to greater humidity loss in both families of polymers. 

In the case of the materials with the highest ionization degrees, the weight losses were around 18% and 20% for the materials PAM-18Na-100 and PAM-18K-100, respectively. In the case of PAM-18Na-75 and PAM-18K-75, the humidity loss was less than 14%, while for the materials with the lowest ionization degrees, the losses were between 12% and 10%. These humidity losses are higher than those obtained at temperatures of 100 and 130 °C, and are strongly correlated to those previously observed by the DSC and TGA analyses. These results indicate that the polymeric salts derived from PAM-18 effectively incorporated water and correspond to hydrated polymeric forms. Finally, it is important to mention that the weight losses were not due to thermolysis processes; FT-IR spectra were taken before and after each assay, and no changes were observed between these spectra.

With respect to the results of humidity gain, it was observed that these were very low (<2%), and after the first hour the humidity gain was practically constant (except PAM-18K-100), suggesting that the polymeric salts derived from PAM-18 do not exhibit high hygroscopicity. Additionally, the results showed that an increase in the ionization degree led to a slight rise in the capability to gain humidity. In the case of PAM-18Na-100 and PAM-18K-100 materials, the humidity gain values were approximately 0.6% and 1.8%, respectively, while for the rest of the polymers they were less than 0.6%. In the case of the PAM-18 precursor material, no humidity gain was observed.

### 2.5. Surface Polymer Characterization

**Figure 8:**
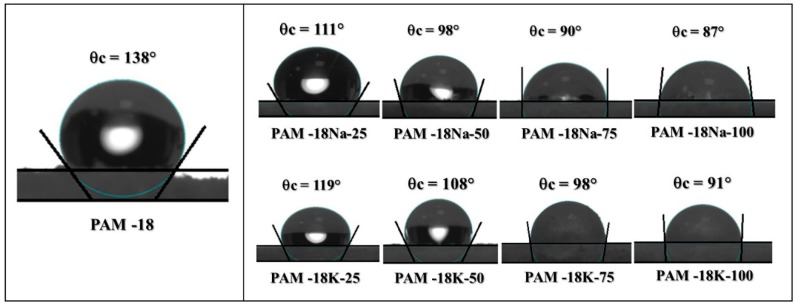
Contact angle variation between ultra-pure water and polymer derived from PAM-18 with different ionization degrees.

In order to know more about effect of the interactions in the humidity gain phenomena, we measured the contact angle (θc) formed between each material and ultra-pure water. The results are shown in Figure 8. These results are very interesting, since they showed that the hydrophobicity is inversely proportional to the ionization degree. Likewise, it was observed that θc values of PAM-18Na were slightly smaller than those of the PAM-18K polymers. In the case of the PAM-18 precursor polymer, a θc value of 138° was observed, suggesting that the polymeric surface is strongly hydrophobic, and this effect is probably given by the specific orientation of the polymer alkyl chains, which are probably pointing normal to the surface. These results are strongly related to those observed in the powder fluidity and humidity gain studies. In the case of PAM-18Na-25 and PAM-18K-25, the θc values were 111° and 119°, respectively, having a very similar behavior to the precursor polymer. In the cases of PAM-18Na-50 and PAM-18K-50, the θc values were 98° and 108°, respectively. On the other hand, for the PAM-18Na-100, PAM-18K-100, PAM-18Na-75, and PAM-18K-75 materials, the θc values were 87°, 91°, 90° and 98°, respectively. These values are close to the “limit zone”, where the wettability phenomenon begins to occur (<90°). These results are consistent if they are related to those previously described, as (i) formation of polymer solutions, (ii) higher ionization performance, (iii) longer lyophilization time, (iv) higher capacity to imbibe water in the form of hydrated polymers, and (v) lower capacity to flow.

## 3. Materials and Methods

### 3.1. Materials

The polymeric precursor material was poly(maleic anhydride-*alt*-octadecene) with molecular weight about of 30–50 kDa (Sigma-Aldrich, St. Louis, MO, USA). NaOH and KOH were obtained from Merck KGaA, Darmstadt, Germany. Type II water was obtained from a purification system (Millipore Elix essential, Merck KGaA, Darmstadt, Germany).

### 3.2. Obtention of Polymeric Materials Derived from PAM-18 

The polymers derived from PAM-18 with different ionization degrees and counterions were produced and characterized according to previously described methods [4,11,20]. Briefly, 100 g of PAM-18 dissolved in 2 L of ultrapure water was mixed with different equimolar amounts of NaOH and KOH (1, 0.75, 0.5, and 0.25). According to the precursor copolymeric unit used, the polymeric materials obtained were named as PAM-18Na and PAM-18K, respectively. The modification was carried out at 40 °C for 24 h under moderate agitation (200 rpm). Subsequently, the polymer solutions were partially evaporated, and the excess of unreacted inorganic-base was removed using stirred ultrafiltration cells 8400 (Amicon®, Merk-Millipore, Billerica, MA, USA) with a 12-kDa cut-off polyethersulfone (PES) membrane. The polymer solutions were lyophilized (model FDU 1110, Eyela, Tokyo Rikakikai, Tokyo, Japan) until obtaining solid materials, which were sieved with 75 μm mesh (number 200). 

### 3.3. Determination of Ionization Degree and Zeta-Potential

The ionization degree was determined by potentiometric acid-base titration, using a pH-meter coupled with a glass-body calomel electrode (Denver Instruments, Bohemia, NY, USA) according to USP methodologies [28]. For this, 0.125 g of polymer was dissolved in 1 L of ultrapure water, which was stirred at 550 rpm for 2 h at 80 °C. Then, each polymer system was titrated with 50 mL 2.63 mM HCl at 25 °C. On the other hand, the zeta potential measurements in aqueous media were performed using a Zetasizer Nano ZSP (Malvern Instrument, Malvern, UK) at 25 °C with a disposable folded capillary cell (DTS1070). 

### 3.4. Structural Characterizations of Polymeric Materials 

#### 3.4.1. FT-IR Characterization

The structural characterization of polymeric materials was performed in an infrared spectrometer (Nicolet 6700, Thermo Fisher Scientific, Waltham, MA, USA), where spectral signals of the precursor material, PAM-18, PAM-18Na, and PAM-18K were compared.

#### 3.4.2. X-ray Diffraction (XRD) 

The X-ray diffraction patterns were obtained on an empyrean diffractometer (XPERT Panalytica, Serie II, detector PIXCel3D, 2012, operated at 40 kV) equipped with a monochromatic CuKα (α_1_ = 1.5406 Å; α_2_ = 1.5443 Å).

#### 3.4.3. Thermal Analysis

Thermal studies were carried out on a Q2000 differential scanning calorimeter (DSC; TA Instruments, New Castle, DE, USA) calibrated with indium T_m_ = 155.78 °C, ∆H_m_ = 28.71 J/g. Thus, three heating–cooling cycles from −60 °C (183.15 K) to 250 °C (523.15 K) with a heating rate of 10 °C/min were applied. The thermogravimetric study was conducted on a TGA instrument (HIRES TGA 2950, TA Instruments, New Castle, DE, USA) at a heating rate of 10 °C/min under a nitrogen atmosphere from 25 °C to 800 °C. Approximately 20 mg of powder was placed on a crucible without a lid, and an empty crucible without a lid was used as a reference.

### 3.5. Characterization of Powder Polymeric Materials 

#### 3.5.1. External Morphology Description

External morphologies of the PAM-18Na and PAM-18K polymers with different ionization degree were observed by Scanning Electron Microscopy (SEMPro, PhenomWorld, Eindhoven, The Netherlands). 

#### 3.5.2. Particle Characterization

Two powder parameters were used, namely the angle of repose and the Carr and Hausner indexes [29,30]. These parameters show the capability of the powder to flow. The flowability degree was obtained through a powder flow tester (Erweka GmbH, Heusenstamm, Germany), while the percentage of compressibility was determined using a density meter (Logan Tap-2S, Logan Instrument, Somerset, NJ, USA) [31]. Briefly, 50 g of the polymer was dried at 100 °C until reaching a constant weight.

#### 3.5.3. Humidity Loss and Gain Studies

The humidity gain was determined using 2 g of polymer, which was previously dried at 120 °C for 24 h. Then, each polymeric material was taken to a stability chamber (40 °C ± 5 °C and relative humidity of 75% ± 2%), where weight measurements were made until reaching a constant value. With respect to the humidity loss, 2 g of polymer was also used, but in this case, a humidity balance (Ohaus MB35) was used with a temperature range between 100 °C and 160 °C.

### 3.6. Polymeric Surface Characterization

First, the powder polymers were compressed using a homemade tableting machine with ¼ inch stainless steel flat punches. For each tablet, 300 mg of polymeric material was used, and a generic pressure of 400 psi was applied for 10 s. Once the compact surfaces of the polymeric materials were formed, the static contact angle was measured. For each system, the sessile drop method was employed using a contact angle meter (OCA15EC Dataphysics Instruments, Filderstadt, Germany) with software controller (SCA20 version 4.5.14). The data capture was recorded using an IDS video camera, where the information obtained was taken in a range of 400–800 frames as a reference point. Moreover, the contact angle capture point was defined when the reflection of the incident drop light disappeared completely (approximately 1 s, since its exit from the dispensing system). A fixed height for dropping of 1 cm was taken. The drop volumes were found in a range of 0.005–0.015 mL. Each measurement was performed at 22 °C ± 1 °C temperature and at a relative humidity of 60% ± 5%, determined with a generic digital Thermo hygrometer. The contact angle was measured in triplicate over the surface of the tablets using type II water. 

## 4. Conclusions

The polymeric materials derived from PAM-18 showed reaction yields greater than 90% for each case studied. Nevertheless, we obtained an ionization degree near to that would be expected when a polymer and an inorganic base are used in a molar ratio of 1:1. In the case of the rest of the polymeric materials, the ionization degree reached was lower. The changes in the FT-IR signals from ~1773 and 1704 cm^−1^ to ~1706 and 1556 cm^−1^, as well as the pH and zeta potential values, corroborated the formation of ionic groups corresponding to carboxylate in the polymeric backbone, which decreased as the proportion of inorganic base used in the polymer modification process was lower. The XRD results showed that the polymer modification process did not affect the tacticity and amorphous characteristics of the precursor material, while the results of the thermal studies showed that such materials had a high capacity to bind water to the polymer structure and that it was greater as the degree of ionization in the polymer increased. Each of the polymeric materials obtained showed flowability from intermediate to low, due to the morphology of irregular plates obtained by the lyophilization process. On the other hand, the results of contact angle measurements showed that the ionization degree affected the characteristics of the polymeric surfaces, where the hydrophobicity of the polymeric surface increased with a decrease in ionization degree.

## Figures and Tables

**Table 1:** Ionization degree obtained for polymeric materials derived from PAM-18.

Polymeric System	Molar Ratio Polymer: Base	Polymer Ionization Degree (%)		pH ± SD		Zeta-Potential ± SD	
		PAM-18Na	PAM-18K	PAM-18Na	PAM-18K	PAM-18Na	PAM-18K
PAM-18M-100	1:1	95	99	11.2 ± 0.0	11.1 ± 0.0	−59 ± 0.9	−58.5 ± 0.1
PAM-18M-75	1:0.75	63	52	10.6 ± 0.1	10.5 ± 0.0	−49.3 ± 1.3	−54.7 ± 3.3
PAM-18M-50	1:0.5	39	35	8.6 ± 0.1	8.3 ± 0.0	−37.7 ± 1.0	−44.1 ± 1.6
PAM-18M-25	1:0.25	22	20	6.7 ± 0.1	6.5 ± 0.1	−37.9 ± 0.3	−39.1 ± 2.1

M: corresponds to the type of counter-ion associated with the polymeric material (Na^+^ or K^+^).

**Table 2:** Flowability data for polymeric material derived from PAM-18.

Polymer Material	Repose Angle (°) ± SD	Carr Index ± SD	Hausner Index ± SD
PAM-18	28 ± 0.5	20.7 ± 1.3	1.3 ± 0
PAM-18Na-25	37.4 ± 1.3	22.6 ± 0	1.3 ± 0
PAM-18Na-50	35.2 ± 0.6	18 ± 0	1.2 ± 0
PAM-18Na-75	33.3 ± 1.1	17.8 ± 0	1.2 ± 0
PAM-18Na-100	30.5 ± 1.6	16.9 ± 1	1.2 ± 0
PAM-18K-25	45.1 ± 1	20 ± 0	1.3 ± 0
PAM-18K-50	43 ± 1.4	16.1 ± 0	1.2 ± 0
PAM-18K-75	39 ± 0.7	15.4 ± 0	1.2 ± 0
PAM-18K-100	38.1 ± 1	11.6 ± 1.4	1.2 ± 0
